# Identifying dose constraints for the parotid ducts to minimize patient‐reported xerostomia: Is conventional mean dose sparing of the parotid glands sufficient?

**DOI:** 10.1002/acm2.14515

**Published:** 2024-09-17

**Authors:** Manal Ahmidouch, Shiva K. Das, Tong Zhu, Colette Shen, Lawrence B. Marks, Bhishamjit S. Chera, David V. Fried

**Affiliations:** ^1^ University of North Carolina at Chapel Hill Chapel Hill North Carolina USA; ^2^ Department of Radiation Oncology University of North Carolina at Chapel Hill Chapel Hill North Carolina USA; ^3^ Department of Radiation Oncology Washington University at St. Louis St. Louis Missouri USA; ^4^ Department of Radiation Oncology Medical University of South Carolina Charleston South Carolina USA

**Keywords:** dose constraint, IMRT, parotid, xerostomia

## Abstract

**Background and purpose:**

The aim of this study was to identify dose constraints for the parotid ducts that limit patient‐reported xerostomia and estimate whether these constraints are achieved during conventional parotid gland sparing radiation therapy (PGS‐RT).

**Methods and materials:**

Thirty‐eight oropharyngeal squamous cell carcinoma patients were treated prospectively on trial with MRI sialography‐guided parotid duct sparing radiation therapy (PDS‐RT). PDS‐RT explicitly minimizes dose to the parotid ducts in addition to PGS‐RT. Parotid duct dose constraints were identified that distinguished patients reporting high and low rates of xerostomia. Atlas‐based parotid duct contours were generated on a retrospective cohort of similar patients where the parotid ducts were not contoured nor explicitly spared to estimate the dose received by the parotid ducts during PGS‐RT.

**Results:**

Patients whose intraglandular parotid ducts or total parotid ducts were planned for a mean dose < 14 Gy and < 12 Gy, respectively, reported significantly (*p* < 0.01) lower rates of xerostomia at 6 and 12 months post‐RT. Patients receiving PDS‐RT had average total and intraglandular duct doses of 11.6  and 13.6 Gy, respectively, compared to an estimated 23.8  and 22.1 Gy, for those receiving PGS‐RT (*p* < 0.01). Only 6% (6/108) and 20% (22/108) of patients receiving PGS‐RT were estimated to meet the dose constraints for the total ducts and intraglandular ducts, respectively.

**Conclusion:**

Parotid duct dose thresholds exist that appear to distinguish patients with and without xerostomia. The identified dose thresholds are frequently not met in PGS‐RT plans. In addition to reducing the dose to the parotid gland(s), parotid duct sparing may also further reduce xerostomia.

## 1 INTRODUCTION

Head and neck cancers account for nearly 3% (∼54 000 new diagnoses each year) of all cancers in the United States.^1^ Radiation therapy (RT) is a mainstay in the treatment of head and neck cancer and is often used with concurrent or sequential chemotherapy and/or surgery. Malignancies in this area are frequently close to vital normal tissue structures and definitive RT treatments often result in substantial morbidity. One of the most common and prominent toxicities after head and neck RT is radiation‐induced xerostomia (dry mouth). These toxicities lessen a patient's ability to adequately chew, swallow, speak, and dramatically reduce many aspects of oral and dental health leading to malnutrition and weight loss. Xerostomia symptoms can persist anywhere from 6 months to many years after treatment and many patients’ functions never return to baseline.^2^, ^3^, ^4^ This extensive symptom burden has been repeatedly shown to significantly decrease quality of life post‐RT, worsen with time, and has a larger effect on younger patients.^5^, ^6^

It has been observed that salivary glands, specifically the parotid glands, have a regional dose dependence. Emerging evidence has supported the hypothesis that stem/progenitor cells capable of gland repair may preferentially reside in the major salivary ducts and could be driving this regionally dependent dose response.^7^, ^8^, ^9^, ^10^, ^11^ Preclinical data alongside retrospective assessments have suggested that the area of the major salivary ducts is more correlated to gland injury and recovery than other areas of the parotid gland(s).^12^, ^13^ This concept, although different mechanistically, is similar to what has been demonstrated in the brain with hippocampal sparing where reducing the dose to this critical region has led to reductions in patient toxicity.^14^ *A prospective trial previously conducted by our group demonstrated that patients undergoing MRI‐guided parotid duct sparing radiation therapy (PDS‐RT) had improved patient reported xerostomia compared to a historical cohort that underwent conventional parotid gland sparing radiation therapy (PGS‐RT)*. MRI guidance (MRI sialography) is needed in order to adequately visualize the parotid ducts as they are not visible on conventional CT.^15^ PDS‐RT is an extension of PGS‐RT whereby the parotid ducts are prospectively identified (in our case via MRI sialography) and spared during treatment planning. The parotid ducts are planned to receive as low of a dose as possible *in addition* to sparing the parotid glands themselves. Further, that trial demonstrated that the dose to the bilateral parotid *ducts* was significantly associated with patient reported xerostomia even when controlling for the dose delivered to the bilateral parotid *glands* and contralateral submandibular gland. This trial highlighted the potential importance of sparing the parotid *ducts* to minimize patient xerostomia.

Currently, there are no accepted dose constraints for the parotid ducts nor any data quantifying the improvement in parotid duct dose sparing achieved between those receiving PDS‐RT versus PGS‐RT. We hypothesize that the dose received by the parotid ducts during PGS‐RT is too high to minimize the rates of xerostomia compared to patients receiving PDS‐RT. Therefore, this work aimed to (1) determine a dose constraint for the parotid *ducts* that would reasonably stratify patients with and without patient‐reported xerostomia, and (2) estimate parotid duct doses via atlas‐based segmentation in a retrospective cohort of patients who did not receive MRI sialograms and underwent PGS‐RT. Aim 1 would provide a dosimetric constraint, which could be used when conducting future PDS‐RT trials and Aim 2 would allow for a comparison between PDS‐RT and PGS‐RT in regards to parotid duct dosimetry to determine whether the aforementioned parotid duct constraint is routinely met using PGS‐RT. The purpose of this work is not to determine whether PDS‐RT yields superior xerostomia outcomes compared to PGS‐RT, but to quantify important dosimetric measures, such as parotid duct dose constraints, which will facilitate prospective trials examining PDS‐RT moving forward.

## 2 METHODS AND MATERIALS

### 2.1 Patient cohorts

Two previously treated patient cohorts were retrospectively reviewed. First, all 38 patients who were treated with definitive RT for oropharyngeal squamous cell carcinoma on our PDS‐RT prospective trial (NCT XXXXXX) were reanalyzed (PDS‐RT Cohort). Twenty‐eight of 38 of these patients were treated to 60 Gy to the high‐risk planning target volume with the other 10 patients treated to 70 Gy. Second, an IRB approved (IRB XX‐XXXX) comparison cohort of 108 patients treated at our institution between 2012 and 2019 with definitive PGS‐RT delivering 60 Gy to the high‐risk planning target volume for oropharyngeal squamous cell carcinoma were identified (PGS‐RT Cohort). The PGS‐RT cohort did not have patient‐reported outcomes available. All patients were staged using the AJCC 7^th^ edition staging system. Table 1 shows the pertinent patient and treatment characteristics of both cohorts.

**TABLE 1:** Patient characteristics.

	PDS‐RT cohort (*n* = 38)	%	PGS‐RT cohort (*n* = 108)	%	*p*
T Stage (7^th^ edition)					0.003
T0‐T1	11	29	37	34	
T2‐T3	23	61	71	66	
T4	4	10	0	0	
N Stage (7^th^ edition)					0.12
N0‐N2a	7	18	23	21	
N2b	22	58	67	62	
N2c	7	18	18	17	
N3	2	6	0	0	
Prescription					NA
60 Gy to PTV high risk	28	74	108	100	
70 Gy to PTV high risk	10	26	0	0	
Median PTV high Risk volume (ccs)	169.5	NA	166.1	NA	0.91
Median PTV standard risk volume (ccs)	880.5	NA	800.6	NA	0.02
Mean parotid dose (Gy) (Median) [SD]	27.2 (27.8) [5.7]	NA	31.4 (31.5) [6.4]	NA	0.001

### 2.2 MRI sialography, parotid duct contouring, and dose sparing

For the PGS‐RT cohort, only the mean dose to the parotid gland(s) was optimized during treatment planning. For the PDS‐RT cohort, the parotid glands *as well as* the parotid ducts were optimized during treatment planning (i.e., PDS‐RT is when patients undergo PGS‐RT with the additional optimization constraint of minimizing the dose to the parotid ducts). The details regarding the parotid duct localization via MRI sialography and treatment planning for the parotid ductal sparing cohort are provided in our previous work.^15^ In brief, patients underwent CT simulation as per clinical protocol (with their neck in a neutral position with a thermoplastic mask placed for immobilization). The treating physicians delineated the patient's target volumes blinded to the MRI images. Once the target volumes were completed, the MRI sialograms were rigidly registered to the patient's CT simulation scan focusing on the mandible adjacent to the parotid gland(s). The entire extent of the parotid ducts (bilateral) was delineated on the Half‐Fourier single‐short turbo spin‐Echo (HASTE) MRI sequence (MRI sialogram). The parotid ductal contours were given a 2 mm isotropic expansion in order to define a planning risk volume (PRV) for treatment planning. These expanded parotid ductal contours were transferred to the patient's simulation CT scan using the rigid registration matrix. The parotid duct PRVs for the entire duct (not just the intraglandular portion) were maximally spared using Sun Nuclear's FeasibilityDVH, which provides DVH guidance based on an ideal dose distribution.^16^, ^17^ The parotid duct PRV, comprised of either the bilateral total parotid ducts or the bilateral intraglandular portion, was used for all dosimetric assessments. We analyzed both the intraglandular portion of the parotid ducts and the total parotid ducts in our analyses. Distinguishing the impact of the intraglandular portion versus the entire duct would require data from far more than 38 patients, thus we completed our analysis using both for completeness. All patients in both cohorts were treated with volumetric‐modulated arc therapy (VMAT) or tomotherapy.

### 2.3 Patient‐reported symptoms

Patients in the PDS‐RT cohort submitted patient reported symptom questionnaires at both 6 and 12‐month timepoints post‐RT. The primary endpoint of this work was the xerostomia‐specific question from the patient‐reported outcome (PRO) Common Terminology Criteria for Adverse Events (CTCAE) questionnaire that states: “During the past week: What was the SEVERITY of your DRY MOUTH at its WORST?”; with ratings (0) none to (4) very severe. Xerostomia as an endpoint was used, as it was found to be one of the most common and severe symptoms in our previous analyses.^2^ Responses were dichotomized such that a PRO‐CTCAE rating ≥ moderate (score 2−4) qualified as a patient‐reported toxicity, and a rating < moderate (score 0−1) qualified as no toxicity. This dichotomization was done, as it was found to be commonly applied in the literature.^12^, ^15^, ^18^ All patients in PDS‐RT cohort were required to have no xerostomia at baseline as graded by their treating physician using CTCAE.

### 2.4 Atlas‐based segmentation assessment and application

We compiled an atlas using CT images from 24 of 38 patients in the PDS‐RT cohort who received an MRI sialogram prior to undergoing parotid ductal sparing and had their parotid ducts contoured. We used a PRV generated from the MRI sialogram of the entire parotid duct with a 2 mm isometric expansion for assessment, as this was the contour used for treatment planning in the previous study. This PRV was then transferred to each patients CT scan using a rigid registration. These CT scans containing the transferred parotid duct PRV were used as our atlas. To evaluate the performance of the atlas‐based segmentation, we applied the atlas to the remaining 14 of 38 patients who did not contribute to the atlas using Raystation v8.99. Fifteen fusion atlases were considered when applying the atlas meaning that the 15 most similar atlas patients were deformably fused to each remaining patient, and the results were then merged to determine a singular result. Using this 14‐patient validation set, we compared the atlas‐based duct contours to the contours transferred onto their planning CT from their patient‐specific MRI sialograms. The mean distance to agreement and distribution of dose differences between the two duct contours (atlas‐based and MRI sialography‐based) were used to assess for overall accuracy of the atlas‐based segmentation—see Statistical Analysis section. This atlas was then applied to the 108 patients in the conventional cohort to estimate their parotid duct doses, as they did not receive MRI sialograms.

### 2.5 Statistical analysis

Pearson's Chi‐squared tests with Yates' continuity correction were used for evaluating the independence of patient characteristics between cohorts. Two‐sided Wilcoxon Rank Sum tests were used to compare dosimetric and planning target volumes values between cohorts. Parotid duct dose constraints were identified by the mean dose value of the bilateral parotid ducts that maximally separated the proportion of patients with and without toxicity (by minimizing the resulting p‐value) when assessing both 6 and 12‐month timepoints via one‐sided Fisher exact tests at 1 Gy intervals (requiring a minimum of 10 patients per group). Both the total parotid duct and the intraglandular parotid duct were assessed. R version 3.6.3 was used for all analyses.

The mean distance to agreement comparing the atlas‐based duct contours compared to the MRI‐defined duct contours was calculated within Raystation v8.99. To estimate the specificity of the atlas‐based duct segmentation with respect to the defined duct constraints, the mean and standard deviation of the dose differences between the MRI‐defined and atlas‐defined duct doses in the 14 patient validation cohort were determined. The computed mean and standard deviation were then used to define a normal distribution, which was sampled and added back to the MRI duct doses for the 38 patients in the PDS‐RT cohort. This estimated the expected duct doses if one were to use atlas‐based duct contours. This was performed 100 separate times yielding 3800 estimates for analysis. The sensitivity and specificity of the 3800 atlas‐based estimates was determined with regard to meeting (positive) or not meeting (negative) the identified parotid duct constraints.

## 3 RESULTS

### 3.1 Parotid duct constraints

**FIGURE 1:**
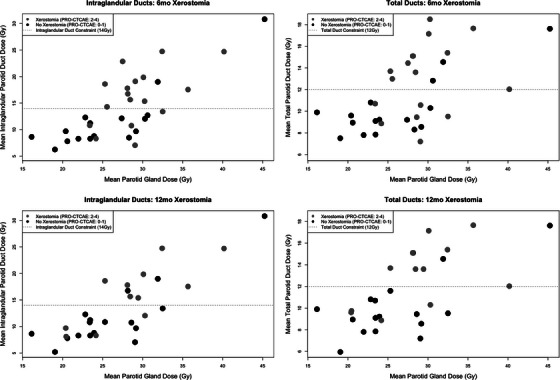
Parotid duct versus parotid gland dose for patient receiving PDS‐RT.

Plots comparing mean parotid duct doses (for both the total ducts and the intraglandular ducts) versus parotid gland mean dose in relation to patient‐reported xerostomia for the PDS‐RT cohort are shown in Figure 1. A cut‐point of 14 Gy to the *intraglandular* parotid ducts, and 12 Gy to the *total* parotid ducts, was best able to distinguish patients with and without xerostomia at both 6 and 12‐month post‐RT (horizontal dashed lines added for reference). Tables 2 and 3 summarize the incidence of toxicity in the parotid duct sparing cohort with respect to the parotid duct constraints for both 6 and 12‐month post‐RT. Xerostomia rates in patients whose parotid ducts received doses above the defined cut‐points were significantly higher than patients whose parotid ducts received doses below the defined cut‐points at both 6 and 12‐month post‐RT (*p* < 0.01).

**TABLE 2:** Impact of meeting the INTRAGLANDULAR (IG) parotid duct dose constraint in the parotid duct sparing cohort with respect to patient‐reported xerostomia.

	Parotid duct sparing cohort	
	IG Ducts < 14 Gy	IG Ducts > 14 Gy
Mean bilateral parotid gland dose (Gy)	24.6	31.2
Xerostomia rate: 6 mo (PRO‐CTCAE: 2‐4)	26% (5/19)	86% (12/14)
Xerostomia rate: 12 mo (PRO‐CTCAE: 2‐4)	22% (4/18)	73% (8/11)

**TABLE 3:** Impact of meeting the TOTAL parotid duct dose constraint in the parotid duct sparing cohort with respect to patient‐reported xerostomia.

	Parotid duct sparing cohort	
	Total ducts < 12 Gy	Total ducts > 12 Gy
Mean bilateral parotid gland dose (Gy)	24.5	30.9
Xerostomia rate: 6 mo (PRO‐CTCAE: 2‐4)	32% (6/19)	79% (11/14)
Xerostomia rate: 12 mo (PRO‐CTCAE: 2‐4)	22% (4/18)	73% (8/11)

Meeting the parotid duct constraint for the intraglandular ducts or the total ducts was associated with an ∼50% lower absolute rate of patient‐reported xerostomia at 6 and 12 months post‐RT. In addition, most patients undergoing parotid ductal sparing were able to meet the identified constraints. We observed that 59% (22/38) patients and 61% (23/38) patients were able to meet the total ducts < 12 Gy constraint and the intraglandular duct < 14 Gy constraint, respectively. This ∼60% adherence rate for the parotid duct constraint is higher than previously reported 32% adherence rates for the QUANTEC‐defined dose‐volume constraints for the parotid glands when using PGS‐RT.^18^

### 3.2 Sensitivity and specificity of atlas‐based segmentation of parotid ducts

In order to first establish the accuracy of atlas‐based segmentation approach, 24 of 38 patients in the parotid duct sparing cohort were used to establish the atlas for parotid duct segmentation. In the remaining 14 patients used to validate the atlas segmentation, we found the average mean distance to agreement to be 4.5 mm for the total ducts between the atlas‐based and patient‐specific MRI sialography defined parotid duct contours. For the intraglandular ducts, we found the average mean distance to agreement to be 5.1 mm. The distance to agreement and dosimetric data for each validation patient is shown in Table 4.

**TABLE 4:** Results of atlas‐segmentation of the parotid ducts in 14 patient validation.

	Total duct MDA (mm)	Mean dose to total ducts: MRI based (Gy)	Mean dose to total ducts: Atlas based (Gy)	Total ducts dose difference (Gy)	IG duct MDA (mm)	Mean dose to IG ducts: MRI based (Gy)	Mean dose to IG ducts: Atlas based (Gy)	IG ducts dose difference (Gy)
Pt1	5.3	15.1	11.6	3.5	7.4	16.8	9.4	7.4
Pt2	7.9	15.1	13.1	2.0	6.9	17.8	16.1	1.7
Pt3	4.5	9.9	9.2	0.7	4.7	8.6	10.1	−1.5
Pt4	4.9	7.9	8.1	−0.2	3.9	8.3	7.5	0.8
Pt5	3.8	10.3	10.6	−0.3	5.5	12.1	8.0	4.0
Pt6	5.5	14.5	8.7	5.7	7.0	22.9	19.5	3.4
Pt7	5.7	11.6	10.3	1.3	6.0	10.9	12.1	−1.3
Pt8	4.5	10.7	9.2	1.5	5.4	10.8	8.9	1.9
Pt9	3.4	14.6	9.0	5.5	4.2	19.0	19.7	−0.7
Pt10	2.9	18.5	17.2	1.3	3.3	15.4	18.2	−2.8
Pt11	1.6	13.7	13.3	0.4	1.6	18.6	8.4	10.3
Pt12	1.2	9.8	12.4	−2.6	1.3	8.1	7.7	0.4
Pt13	6.5	13.6	14.2	−0.6	8.8	15.4	15.2	0.2
Pt14	4.7	6.0	10.9	−5.0	5.3	5.2	6.2	−1.0
**Average**	***4.5***	**12.2**	**11.3**	***0.96***	***5.1***	**13.5**	**11.9**	***1.6***

Abbreviations: IG, intraglandular; MDA, mean distance to agreement.

The average dose difference and standard deviation between the MRI‐defined total duct contours and atlas‐defined total duct contours was 0.96 Gy and a standard deviation of 2.9 Gy. The average dose difference and standard deviation between the MRI defined intraglandular duct contours and atlas‐defined intraglandular duct contours was 1.6 Gy and a standard deviation of 3.6 Gy. We sampled a normal distribution with the mean and standard deviations and added the values back to each of the parotid duct dose values of 38 patient PDS‐RT cohort. This was done 100 times yielding 3800 parotid duct dose values for analysis. From these 3800 values, we calculated sensitivity and specificity values with respect to meeting the parotid duct dose constraint(s). The sensitivity and specificity of the dose values estimated from the atlas‐based segmentation were 73% and 88% for the total duct and 74% and 92% for the intraglandular duct, respectively. Our hypothesis was that patients getting PGS‐RT would not meet the parotid duct constraints, and therefore, the specificity metric was considered more important (the proportion of patients accurately predicted not to meet the duct constraint). Given the high specificity values of 88% and 92%, we deemed the atlas‐based segmentation of parotid duct dosimetry sufficient to facilitate an estimate of parotid duct dose in patients undergoing standard of care mean dose parotid gland sparing.

### 3.3 Estimated parotid *duct* doses in patients receiving PGS‐RT

The atlas‐based segmentation assessed in the previous section was applied to the PGS‐RT cohort. The estimated mean doses to the total parotid ducts and intraglandular parotid ducts in the PGS‐RT cohort were found to be significantly higher than the parotid duct sparing cohort (23.8  vs. 11.6 Gy for the total ducts and 22.1  vs. 13.6 Gy for the intraglandular parotid ducts; *p* < 0.001). Only 6% (6/108) and 20% (22/108) of treatment plans in the PGS‐RT cohort met the dose constraints for the total ducts and intraglandular ducts, respectively. These percentages were significantly less than the ∼60% rate observed in the PDS‐RT cohort (*p* < 0.001).

## 4 DISCUSSION

We were able to identify parotid duct dose constraints (for both the total ducts and intraglandular portion of the ducts) capable of reasonably stratifying patients at low versus high rates of patient‐reported xerostomia at 6 and 12 months post‐RT. The parotid duct dose constraints were found to have high achievability compared to previous reports in the literature (∼60% vs. 32% of patients) assessing the achievability of the QUANTEC parotid gland dose constraints for those receiving PGS‐RT via IMRT.^18^ Using atlas‐based segmentation to generate estimates of the dose delivered to the parotid ducts during conventional parotid gland mean dose sparing, we found that only 6% and 20% of the conventional mean parotid gland sparing plans met the identified dose constraints for the total ducts and intraglandular ducts, respectively.

**FIGURE 2:**
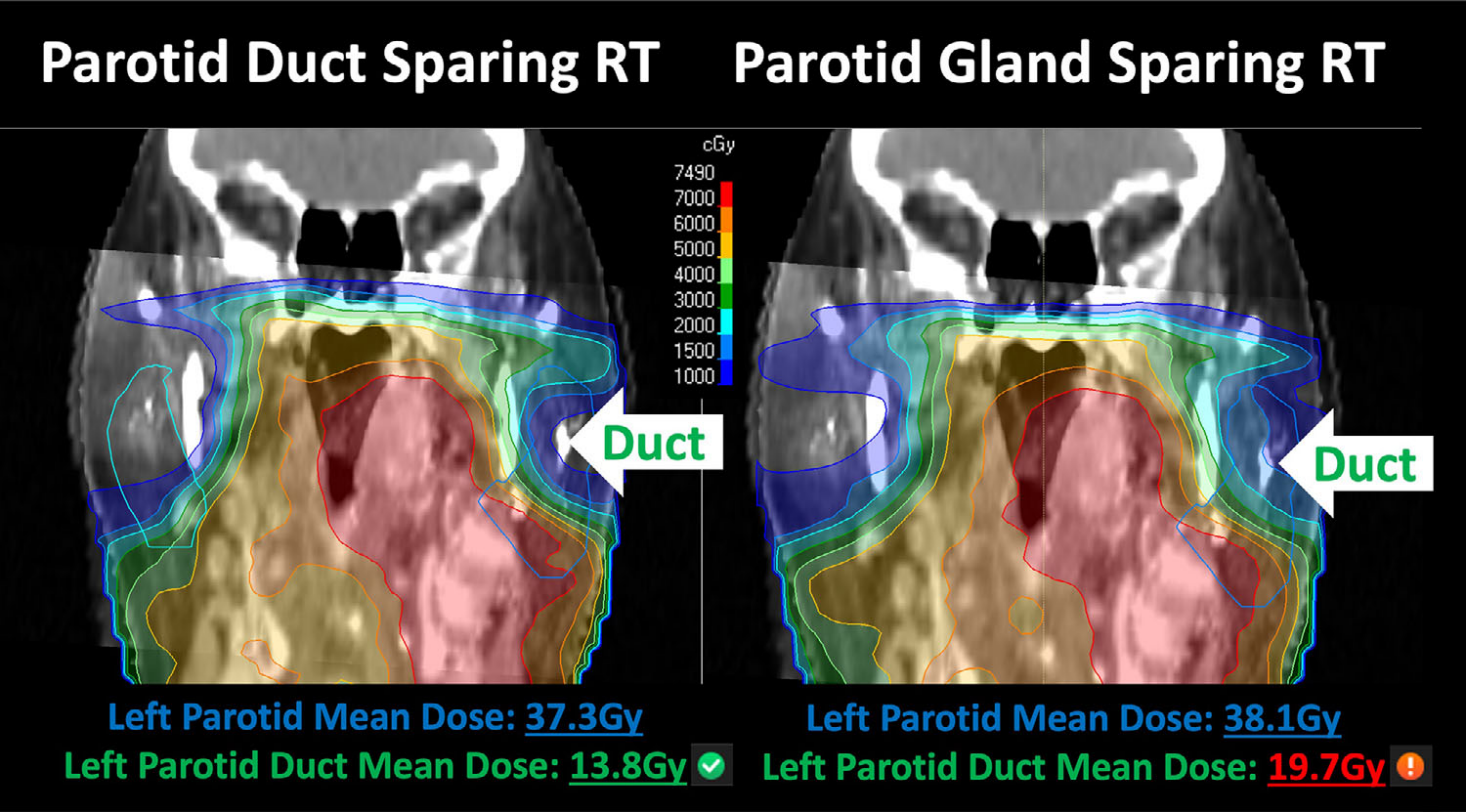
Example plan comparison between PDS‐RT and PGS‐RT.

We hypothesize that parotid duct sparing may be particularly effective since it can be applied to the *bilateral* ducts. In conventional parotid *gland* mean dose sparing, the ipsilateral parotid gland is often not pushed to be spared to the same extent as the contralateral parotid gland due to the proximity of gross disease and higher incidence of failure.^19^ Parotid duct sparing could allow for an effective means of sparing, what our group and others have suggested as perhaps, the “most important” portion of the ipsilateral gland while maintaining coverage of the high‐risk areas. Figure 2 (showing the planned dose on an image fusion of the planning CT and MRI sialogram) illustrates how performing conventional parotid gland mean dose sparing may lead to insufficiently spared parotid ducts (particularly on the ipsilateral side). In this example, a patient from the parotid duct sparing cohort was replanned with conventional parotid gland mean dose sparing. The only difference in the planning was that the constraints on the parotid ducts were excluded during the replan. This led to a comparable mean parotid dose, but a substantially higher dose delivered to the ipsilateral parotid duct—which is consistent to what we observed in our analyses regarding parotid duct doses for PGS‐RT plans.

This work also was able to demonstrate the initial feasibility of using atlas‐based segmentation to identify the parotid ducts and subsequently estimate the dose planned to the parotid duct region for patients undergoing standard of care PGS‐RT. As expected, the dose to the parotid ducts was significantly higher (23.8  vs. 11.6 Gy for the total ducts and 22.1  vs. 13.6 Gy for the intraglandular parotid ducts) for those undergoing PGS‐RT compared to PDS‐RT. While a direct comparison of these dose values is far from perfect, it does support the idea that PDS‐RT substantially reduces the parotid duct doses compared to PGS‐RT. While atlas‐based segmentation was found to be sufficient (based on an ∼90% specificity of maintaining duct constraint result) for our purposes in this work, further study is needed to improve and validate ways to automatically segment the parotid ducts without an MRI sialogram.

While this work was successful in identifying reasonable parotid duct dose constraints, there are several limitations and caveats. First, this is a retrospective study and all caveats pertaining to retrospective analyses apply. Second, all patients in the PGS‐RT cohort were not planned with FeasibilityDVH for gland sparing (this was implemented clinically at our institution in 2016), whereas all patients in the PDS‐RT cohort had the benefit of being planned with FeasibilityDVH. This could be the reason behind patients in the PDS‐RT cohort receiving a lower average parotid gland dose compared to the PGS‐RT cohort (see Table 1). In addition, patients in the PGS‐RT cohort had higher T‐stages and larger standard risk PTVs, which may have contributed to the higher parotid doses. An option would have been to use a subset of our PGS‐RT cohort for analysis; however, we felt this would have limited our numbers to unacceptable level. Third, our primary endpoint was a patient‐reported outcome (xerostomia) and therefore is more subjective and less quantifiable than, say, salivary volume. However, patient‐reported outcomes are increasingly being obtained in oncology trials and their validity has been well documented. Fourth, our patient cohort receiving parotid duct sparing was comprised of only 38 patients, and thus, the rates of xerostomia for those meeting and not meeting our identified dose constraints have a considerable amount of uncertainty. Finally, this work does not address whether the entire parotid duct or just the intraglandular parotid ducts need to be spared in future trials, but it does come to conclusions about reasonable dose constraints for both.

This work is the first to establish dose constraints for the parotid ducts for the purpose of minimizing patient‐reported xerostomia. These dose constraints can be used in future trials to determine whether adequate parotid duct sparing is achieved during treatment planning. Given the retrospective nature of this study, further validation is needed to confirm these results and the importance of the dose planned to the parotid duct region. We established the feasibility and initial accuracy of using atlas‐based segmentation to delineate parotid ducts for patients without MRI sialograms. Our atlas‐based segmentation allowed us to estimate that patients receiving PGS‐RT frequently do not meet the identified dose constraints to the parotid ducts even in the setting of de‐intensified treatment. Therefore, patients could potentially benefit from a parotid duct sparing approach. The atlas‐based segmentation technique could be used in the future not only to facilitate dosimetric comparisons but also to potentially enable parotid duct sparing for patients without the need for an MRI sialogram. Additional methods of obtaining accurate parotid duct contours without the need for MRI such as model‐based approaches, machine learning, etc., should be investigated.

The purpose of this work was not to determine whether PDS‐RT yields superior xerostomia outcomes compared to PGS‐RT, but to quantify important dosimetric measures, such as parotid duct dose constraints, that will facilitate prospective trials examining PDS‐RT moving forward. Assessing and utilizing atlas‐based segmentation of the parotid ducts not only enabled estimation of parotid duct doses for patients undergoing PGS‐RT, but it represents a starting point for potentially performing PDS‐RT without acquiring an MRI sialogram, which would allow for broader utilization of PDS‐RT.

## AUTHOR CONTRIBUTIONS

Manal Ahmidouch contributed to this manuscript in terms of design of work, data acquisition and interpretation, drafting, and final approval of version to be published and is accountable for all aspects of the work and ensuring questions relating to the accuracy and integrity of the work are appropriately investigated and resolved. Shiva K. Das contributed to this manuscript in terms of design of work, data acquisition and interpretation, drafting, and final approval of version to be published and is accountable for all aspects of the work and ensuring questions relating to the accuracy and integrity of the work are appropriately investigated and resolved. Tong Zhu contributed to this manuscript in terms of design of work, data acquisition and interpretation, drafting, and final approval of version to be published and is accountable for all aspects of the work and ensuring questions relating to the accuracy and integrity of the work are appropriately investigated and resolved. Colette Shen contributed to this manuscript in terms of design of work, data acquisition and interpretation, drafting, and final approval of version to be published and is accountable for all aspects of the work and ensuring questions relating to the accuracy and integrity of the work are appropriately investigated and resolved. Lawrence B. Marks contributed to this manuscript in terms of design of work, data acquisition and interpretation, drafting, and final approval of version to be published and is accountable for all aspects of the work and ensuring questions relating to the accuracy and integrity of the work are appropriately investigated and resolved. Bhishamjit S. Chera contributed to this manuscript in terms of design of work, data acquisition and interpretation, drafting, and final approval of version to be published and is accountable for all aspects of the work and ensuring questions relating to the accuracy and integrity of the work are appropriately investigated and resolved. David V. Fried contributed to this manuscript in terms of design of work, data acquisition and interpretation, drafting, and final approval of version to be published and is accountable for all aspects of the work and ensuring questions relating to the accuracy and integrity of the work are appropriately investigated and resolved.

## CONFLICT OF INTEREST STATEMENT

The authors declare no conflicts of interest.

## Data Availability

Research data are stored in an institutional repository and will be shared upon request to the corresponding author.
